# A mixed model of heat exchange in stationary honeybee foragers

**DOI:** 10.1038/s41598-023-31320-5

**Published:** 2023-03-21

**Authors:** Anton Stabentheiner, Helmut Kovac

**Affiliations:** Institute of Biology, University of Graz, Universitätsplatz 2, 8010 Graz, Austria

**Keywords:** Ecological modelling, Respiration, Ecophysiology

## Abstract

During foraging honeybees are always endothermic to stay ready for immediate flight and to promote fast exploitation of resources. This means high energetic costs. Since energy turnover of foragers may vary in a broad range, energetic estimations under field conditions have remained uncertain. We developed an advanced model, combining the benefits of mechanistic and correlative models, which enables estimation of the energy turnover of stationary foragers from measurements of body surface temperature, ambient air temperature and global radiation. A comprehensive dataset of simultaneously measured energy turnover (ranging from 4 to 85 mW) and body surface temperature (thorax surface temperature ranging from 33.3 to 45 °C) allowed the direct verification of model accuracy. The model variants enable estimation of the energy turnover of stationary honeybee foragers with high accuracy both in shade and in sunshine, with SD of residuals = 5.7 mW and R^2^ = 0.89. Its prediction accuracy is similar throughout the main range of environmental conditions foragers usually experience, covering any combination of ambient air temperature of 14–38 °C and global radiation of 3–1000 W m^−2^.

## Introduction

**Figure 1:**
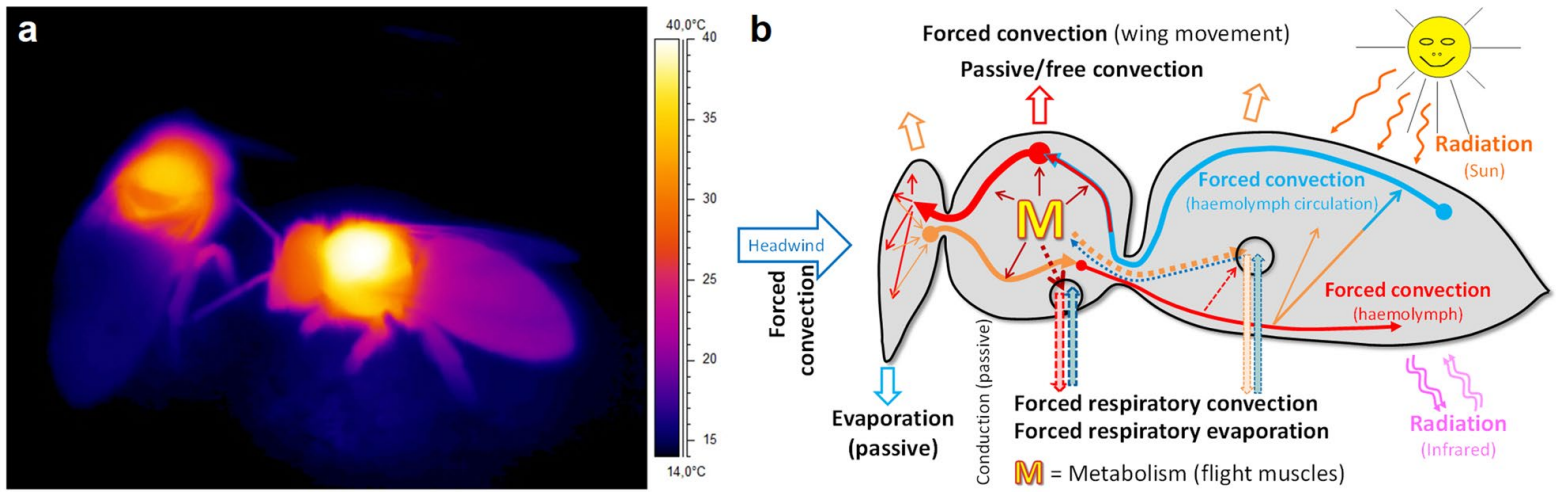
Body temperature and heat exchange of foraging honeybees. (**a**) Infrared thermogram of honeybees (*Apis mellifera carnica*) foraging sucrose solution. Note heated thoraxes resulting from intense endothermy with activated flight muscles. Part of the heat has warmed the head and the abdomen. Ambient air temperature (T_a_) = 13 °C. (**b**) Main paths of heat flow, inside the body and in and out of it. Circles symbolize spiracles (6 on thorax, and 12 on abdomen;^56,57^). M = metabolism, mainly of flight muscles plus standard metabolism of other tissues.

Honeybees are able to exploit nectar and pollen sources fast and efficiently by means of persistent endothermy throughout the foraging cycle (Fig. 1a;^1–8^). Keeping the body temperature high allows for immediate flight on their trips between flowers and increases suction speed ^7,9^.

**Figure 2:**
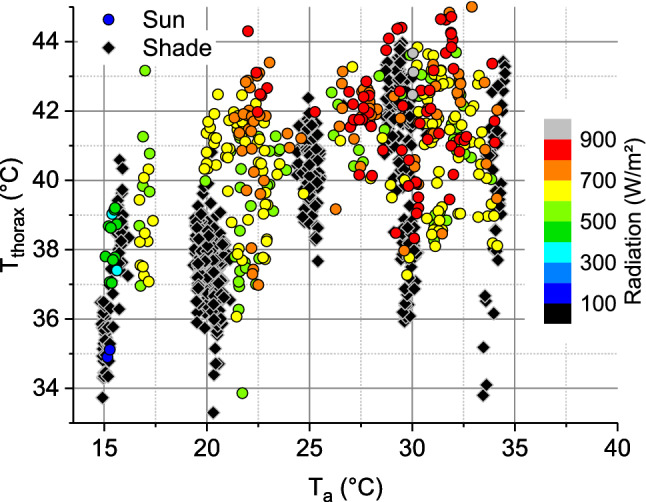
Mean thorax surface temperature per stay at an artificial flower (T_thorax_) in relation to ambient air temperature (T_a_) and global radiation. Shade ≤ 100 W m^-2^, sunshine > 100 W m^−2^. Bees fed 1.5 M sucrose solution in unlimited flow (data from^17^), or 0.5 M sucrose in unlimited flow and limited flow of 15 µl min^−1^ (data from^18^). For simultaneously measured energy turnover see Fig. 3a and supplementary Fig. S2.

**Figure 3:**
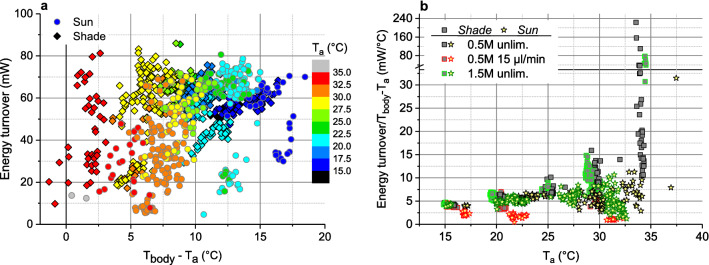
Energetics of honeybees foraging in shade (≤ 100 W m^−2^) or in sunshine (> 100 W m^−2^), from simultaneous measurements of CO_2_ production and body surface temperature (T_body_ = mean of head, thorax and abdomen). (**a**) Energy turnover of sucrose gathering honeybees in dependence on body temperature excess (elevation of mean body temperature per stay above ambient air temperature: T_body_–T_a_). For measured thorax temperatures see Fig. 2. (**b**) Heat conductance estimate from these data in dependence on ambient air temperature (T_a_). Bees fed 1.5 M sucrose in unlimited flow (data from^17^), and 0.5 M sucrose solution in unlimited flow or limited flow of 15 µl min^−1^ (data from^18^).

However, in the endothermic state heterothermic insects of the small size of honeybees have to cope with an enormous heat loss because of their unfavourable relation of surface to mass^1,10^. The challenge is high because environmental conditions like ambient air temperature and solar radiation may vary in a broad range during foraging (Fig. 2)^7,8^. In addition, variation of the quality and availability of food influence thermoregulation of foragers strongly both outside the colony^3,4,11,12^ and inside it^13,14^. Therefore, measurements of metabolic rate (and thus energy turnover) show a large variation in stationary foragers at artificial food sources^15–18^, in (free) flight^19–22^, and in bees flying from flower to flower^23,24^. Because of this strong dependence on environmental and experimental conditions (Figs. 2, 3a; Ref.^22^) the applicability of laboratory measurements for estimations of energy costs under field conditions seems quite uncertain. Inside the honeybee colony estimation of the heat production of endothermic bees is even more problematic. The contribution of individuals to heat production is not accessible by direct respiratory or heat production measurements, because the energy turnover may change by a factor of 5–100 due to changes in endothermy upon transfer of a bee to a respiration measurement chamber^25^.

Therefore, it would be a great advance to have the possibility to estimate the energy turnover of bees from measurements of body temperature and easily accessible environmental parameters like ambient air temperature (T_a_) and solar radiation. Models of heat exchange allowing this may be of a mechanistic or a correlative nature. Mechanistic models of insect heat exchange (e.g.^21,26–28^) rely on detailed knowledge of physical and physiological parameters of heat exchange. Correlative models (e.g.^29–31^) use known relationships without detailed knowledge of all underlying mechanisms^32^.

In previous investigations we had gathered a comprehensive dataset of simultaneous measurements of body surface temperature and respiratory metabolism of stationary foragers under widely varying environmental conditions^17,18^, covering the greater part of the range of natural variation in ambient air temperature and global radiation honeybees usually experience outside their colony. These datasets provided a unique possibility to develop a model of heat exchange between honeybees and their environment and to prove its accuracy. However, in honeybees several parameters of the heat exchange process cannot be determined well enough experimentally. Total convection acting on a foraging bee, for example, is not accessible well enough under true field conditions. While it might be possible to measure wind and free thermal convection directly, the exact heat loss via respiratory convection seems not easily accessible. The absorption of solar radiation and the heat exchange via long-wave infrared radiation is usually modelled by describing the insect body by standard geometrical shapes (e.g.^21,28^; supplementary Fig. S1a). In honeybees, body hairs, wings and variation of absorptivity at visual wavelengths due to changes in body posture influence radiative and convective heat exchange considerably and make estimation uncertain (compare supplementary Fig. S3).

Therefore, we here present a mixed approach of honeybee heat exchange with the environment, combining the benefits of mechanistic and correlative models. Our model variants use detailed physical input wherever available but correlative weighting of parameters by coefficients determined iteratively. The simultaneous measurement of body surface temperature, metabolism and environmental parameters in our dataset allows exact verification of model accuracy.

## Materials and methods

### The basic model

In thermal steady state, total energy gain of an animal equals energy loss (e.g.^33,34^:

1 $${\text{E}}_{{{\text{gain}}}} = {\text{E}}_{{{\text{loss}}}} ;$$

(for abbreviations used see Table 1, iteratively calculated coefficients are written in *italics* there and below). Energy ‘gain’ of a live animal consists of metabolic heat production (M) and radiation heat gain (R_gain_). Energy loss makes up of convective (E_cv_), evaporative (E_ev_), conductive (E_cd_) and radiation heat loss (R_loss_), Eq. (1) taking the form of

2 $${\text{M}} + {\text{R}}_{{{\text{gain}}}} = {\text{R}}_{{{\text{loss}}}} + {\text{E}}_{{{\text{cv}}}} + {\text{E}}_{{{\text{ev}}}} + {\text{E}}_{{{\text{cd}}}} .$$

**Table 1:** Abbreviations or symbols used in formulas and text. Iteratively calculated coefficients are written in *italics* (left column).

Abbreviation/symbol	Meaning	Units	Value/comment
M	Metabolism or Energy turnover	W	Flight muscles + standard metabolism
E_gain_, E_loss_	Energy: gain and loss	W	
R_loss_ = R_lossIR_ = R_lIR_	Radiation heat loss: total = infrared (IR)	W	
*r*_*l*_	Coefficient of radiation heat loss		
R_gain_; R_gSOL_, R_gIR_	Radiation heat *g*ain: total; solar (SOL) and infrared (IR)	W	
*r*_*g*_	Coefficient of radiation heat gain		
R_dSOL_, R_rSOL_	Solar (SOL) heat gain: *d*irect or *r*eflected	W	
R_dIR_, R_rI_, R_geIR_	Infrared (IR) heat gain: *d*irect, *r*eflected or *g*round *e*mitted	W	
α_bSOL_; α_bIR_	Absorption coefficient: solar (SOL) and infrared (IR) radiation		~ 0.825; 0.97 ^12^
α_paint_	Absorption coefficient of measurement chamber interior painting		0.95
ε_b_	Infrared emissivity of the honeybee *b*ody		0.97 ^12^
E_cv_	Convective heat loss	W	
*h*,* h*_*b*_;* h*_*hd*_*, h*_*th*_*, h*_*ab*_;* h*_*thab*_	E_cv_ heat exchange coefficients: general, of *b*ody; of *h*ea*d*, *th*orax, *ab*domen; of *th*orax to *ab*domen	W m^−2^ °C^−1^	Simple; Advanced; Advanced three-compartment model
*a*_*hhd*_, *b*_*hhd*_; *a*_*hth*_,*b*_*hth*_; *a*_*hab*_,*b*_*hab*_	E_cv_ linear heat exchange functions: *a*_*hxx*_ = intercept, *b*_*hxx*_ = slope; of *h*ea*d*, *th*orax, *ab*domen	W m^−2^ °C^−1^, W m^−2^ °C^−2^	Advanced model variants
*a*_*hthhd*_, *b*_*hthhd*_; *a*_*hthab*_, *b*_*hthab*_	E_cv_ linear heat exchange functions: *a*_*hxxyy*_ = intercept, *b*_*hxxyy*_ = slope; of *th*orax to* h*ea*d*, *th*orax to *ab*domen	W m^−2^ °C^−1^, W m^−2^ °C^−2^	Advanced three-compartment model variants
E_ev_	Evaporative heat loss	W	~ 4 mW ^21,22^
*e*_*lev*_	Coefficient of evaporative heat loss		
E_cd_	Conductive heat loss	W	Neglected in models
A_b_; A_hd_, A_th_, A_ab_	Surface area: of total *b*ody; of *h*ea*d*, *th*orax and *ab*domen	m^2^	
T_b_ = T_body_; T_hd_, T_th_, T_ab_	Body (surface) temperature: mean of head, thorax and abdomen; or of *h*ea*d, th*orax, *ab*domen	°C, K	
T_a_	Ambient air temperature	°C	Within ~ 1 cm of the bee
T_b_–T_a_	Body temperature excess above T_a_	°C	
σ	Stefan/Boltzmann constant	W m^−2^ K^−4^	5.669 × 10^–8^
Heat conductance	Energy turnover/(T_b_–T_a_)	W °C^−1^	
Global radiation	Solar radiation	W m^−2^	Visible and infrared
f_resp_	Respiratory frequency of foragers	Hz	
SD_res_	Standard deviation of residuals	mW	
SEM_res_	Standard error of mean of residuals	mW	
R^2^	= adjusted for degrees of freedom (df)		

If metabolic heat production is to be determined from body temperature and environmental data, Eq. (2) can be rewritten as

3 $${\text{M}} = {\text{R}}_{{{\text{loss}}}} - {\text{R}}_{{{\text{gain}}}} + {\text{E}}_{{{\text{cv}}}} + {\text{E}}_{{{\text{ev}}}} + {\text{E}}_{{{\text{cd}}}} \left[ {\text{W}} \right].$$

E_cv_ can be estimated from the temperature difference between insect body (surface) temperature (mean of head, thorax and abdomen) and ambient air temperature (T_b_–T_a_; in °C), body surface area (A_b_; in m^2^), and a convection coefficient (*h*_*b*_; in W m^−2^ °C^−1^):

4 $${\text{E}}_{{{\text{cv}}}} = {\it{\text{h}}_{{\text{b}}}} \times {\text{A}}_{{\text{b}}} \left( {{\text{T}}_{{\text{b}}} - {\text{T}}_{{\text{a}}} } \right).$$

Equation (3) then becomes

5 $${\text{M}} = {\text{R}}_{{{\text{loss}}}}\,{-}\,{\text{R}}_{{{\text{gain}}}} + {\it{\text{h}}_{{\text{b}}}} \times {\text{A}}_{{\text{b}}} \left( {{\text{T}}_{{\text{b}}} - {\text{T}}_{{\text{a}}} } \right) + {\text{E}}_{{{\text{ev}}}} + {\text{E}}_{{{\text{cd}}}} \left[ {\text{W}} \right].$$

Conductive heat transfer (E_cd_), taking place only from the distal leg tips in honeybees, was assumed to be very small in comparison to other parameters of heat exchange and therefore was neglected in further considerations.

To compensate for any uncertainties of radiative and evaporative heat transfer determination, coefficients *r*_*l*_, *r*_*g*_ and *e*_*lev*_ were introduced:

6 $${\text{M}} = {\it{\text{r}}_{{\text{l}}}} \times {\text{R}}_{{{\text{loss}}}} \,{-}\,{\it{\text{r}}_{{\text{g}}}} \times {\text{R}}_{{{\text{gain}}}} + {\text{E}}_{{{\text{cv}}}} + {\it{\text{e}}_{{{\text{lev}}}}} \times {\text{E}}_{{{\text{ev}}}} ;\,{\text{or}}\,{\text{with}}\,{\text{equation}}\,\left( 4 \right)\,{\text{considered:}}$$

7 $${\text{M}} = {\it{\text{r}}_{{\text{l}}}} \times {\text{R}}_{{{\text{loss}}}} \,{-}\,{\it{\text{r}}_{{\text{g}}}} \times {\text{R}}_{{{\text{gain}}}} + {\it{\text{h}}_{{\text{b}}}} \times {\text{A}}_{{\text{b}}} \left( {{\text{T}}_{{\text{b}}} - {\text{T}}_{{\text{a}}} } \right) + {\it{\text{e}}_{{{\text{lev}}}}} \times {\text{E}}_{{{\text{ev}}}} \left[ {\text{W}} \right].$$

Body surface areas of head, thorax and abdomen were calculated assuming spherical dimensions for the thorax (A_th_), and an oblate or prolate rotational ellipsoid for the head (A_hd_) and the abdomen (A_ab_), respectively^35^ (supplementary Fig. S1a).

Evaporative heat loss E_ev_ was estimated as ~ 4 mW from measurements in flying bees at T_a_ below 35 °C^21,22^. We did not consider the changes of E_ev_ with T_a_ these authors measured due to cooling efforts at high T_a_, because our bees’ mouthparts had been wet from drinking at all environmental conditions.

Radiative heat loss at normal environmental temperatures (0–50 °C) takes place in the medium to long infrared range (maximum radiation at 10.61–8.97 µm wavelength, respectively). It can be calculated according to the Stefan/Boltzmann law as

8 $$ {\text{R}}_{{{\text{loss}}}} = {\text{R}}_{{{\text{lossIR}}}} = {\epsilon}_{{\text{b}}} \times {\sigma} \times {\text{T}}_{{\text{b}}}^{4} \times {\text{A}}_{{\text{b}}} ,$$

where T_b_ is the body temperature in Kelvin, ε_b_ is the infrared emissivity of the honeybee cuticle (0.97^12^; compare also Ref.^36–38^), and σ is the Stefan/Boltzmann constant (5.669 × 10^−8^ W m^−2^ K^−4^; Table 1). Reabsorption of the bees’ own radiative heat emission reflected from the ground was neglected due to the high IR absorptivity of the black paint that had covered the measurement chamber (α_paint_ = 0.95; see Fig. 2 in Stabentheiner et al.^39^).

Radiative heat gain consists of infrared heat gain (R_gIR_) according to the Stefan/Boltzmann law, and of solar heat gain (R_gSOL_), mainly taking place in the visible and near infrared range (compare^28,39^):

9 $${\text{R}}_{{{\text{gain}}}} = {\text{R}}_{{{\text{gIR}}}} + {\text{R}}_{{{\text{gSOL}}}} .$$

In the experiments providing the dataset used for model development^17,18^ R_gSOL_ had been measured directly with custom-manufactured miniature thermoelectric global radiation sensors (Ahlborn FLA613GS/Mini spezial^39^).

Infrared (R_gIR_) and solar (R_gSOL_) radiation components absorbed by the honeybee body were assumed to act on half of the body surface area only (A_b_/2), according to

10 $${\text{R}}_{{{\text{gIR}}}} = \upalpha _{{{\text{bIR}}}} \times {\text{R}}_{{{\text{dIR}}}} \times \left( {{\text{A}}_{{\text{b}}} /2} \right) + \upalpha _{{{\text{bIR}}}} \times {\text{R}}_{{{\text{rIR}}}} \times \left( {{\text{A}}_{{\text{b}}} /2} \right) + \upalpha _{{{\text{bIR}}}} \times {\text{R}}_{{{\text{geIR}}}} \times \left( {{\text{A}}_{{\text{b}}} /2} \right),$$

11 $${\text{R}}_{{{\text{gSOL}}}} = \upalpha _{{{\text{bSOL}}}} \times {\text{R}}_{{{\text{dSOL}}}} \times \left( {{\text{A}}_{{\text{b}}} /2} \right) + \upalpha _{{{\text{bSOL}}}} \times {\text{R}}_{{{\text{rSOL}}}} \times \left( {{\text{A}}_{{\text{b}}} /2} \right),$$

where subscript initials ‘d’, ‘r’ and ‘ge’ denominate ‘direct’, ‘reflected’ and ‘ground emitted’ infrared and solar radiation, respectively. Cuticular infrared absorptivity was calculated from emissivity according to α_bIR_ = ε_b_ = 0.97^12^. Solar absorptivity in the visual and near infrared range (α_bSOL_) was estimated as ~ 0.825, from the value of 0.91 reported by Willmer & Unwin^40^ and 0.903 reported by Stupski & Schilder^28^, corrected forreduced absorptivity towards the edges of the curved body surfaces according to a cosine law (see suppl. Figs. S1a and S3).

### Experiments on respiratory frequency (f_resp_) and metabolism

Convective heat loss of living animals is made up of external free convection (in stationary bees) or forced convection (e.g. by headwind and wing movement in flight or by wind), and internal convection due to respiratory gas exchange (Fig. 1b). In stationary, non-flying bees respiratory heat loss has to be assumed to make up the greater part, being a function of respiratory frequency. However, we had not been able to determine the respiratory frequency (f_resp_) from our thermographic recordings in the dataset used for model development^17,18^. An initial hypothesis was that f_resp_ and thus respiratory heat loss might be a function of the bees’ energy turnover because energetically more active bees will need more oxygen.

To determine the correlation between respiratory frequency and energy turnover, therefore, we trained honeybees to forage sucrose solution (0.5 M or 1.5 M) from an artificial flower on an inverted white plastic laboratory cup closer with a ring of holes drilled in its base, which allowed the air pumps to suck fresh air from underneath the flower (see supplementary Fig. S1b)^39^. This way the air stream of 500 ml/min (regulated with Side Trak 840-L mass flow controllers, Sierra Instruments) washed away the air around the bee sucking at a 1 cm higher position. A differential setup with two identical flowers was used, one for measurement of the bees and another one for a reference air stream (parallel measurement mode^39^). Measurement chambers consisted of a glass laboratory funnel attached to a small plastic cylinder, the base of which was attached to an iron spacer ring to fit the chamber to the underlying artificial flower during measurements via pieces of hard disc magnets (suppl. Fig. S1b). The measurement chamber was operated from a distance (∼ 1.5 m) via a rod to prevent the operators from influencing the measurements with their exhaled air. O_2_ consumption was measured with an Oxzilla II oxygen measurement device (Sable Systems, Las Vegas, USA) recalibrated regularly against the outside air. Loss of O_2_ depleted air upon chamber opening was compensated for by calibrations described in Stabentheiner et al.^39^. Data storage and evaluation was done with ExpeData software (Sable Systems). Respiratory frequency was determined from simultaneously recorded video sequences (30 Hz; Canon Power Shot SX200 IS), evaluated with the VLC Media Player.

### Visualisation of the honeybee tracheal system

The honeybee tracheal system, important for respiratory heat exchange via internal convection and evaporation (Fig. 1b), was visualised by means of a micro computer tomograph (µCT) (microCT 40, SCANCO Medical, Bruettisellen, Switzerland). A fresh honeybee was scanned with a resolution of 7 µm, showing abdominal and thoracic air sacs and tracheae.

### Data evaluation and statistics

Our comprehensive dataset allowed for direct comparison of measured values of energy turnover with the values calculated from the measured body surface temperature, ambient air temperature and global radiation, for each visit of the bees to our feeding station. Experimental data evaluation and model calculation was done in Excel (Microsoft) and Origin (OriginLab) software. Accuracy of fit of model variants was compared by calculation of the standard deviation of residuals (SD_res_) and R^2^ adjusted for degrees of freedom (df).

The fit was optimized by iterative variation of coefficients according to the nonlinear least square Levenberg–Marquardt (L–M) algorithm, an “iterative procedure which combines the Gauss–Newton method and the steepest descent method”^41^. Optimum fit, as estimated by Chi^2^ minimization (residual sum of square divided by df), was assumed when a reduced Chi^2^ tolerance value of 1 × 10^–9^ was reached^41^.

## Results

### Body surface

Body surface area (without wings and legs), as calculated by equations of suppl. Fig. S1a^35^, amounted to A_b_ = 163.7 mm^2^, with A_hd_ = 30.80 mm^2^, A_th_ = 47.78 mm^2^, and A_ab_ = 85.91 mm^2^ (for measured body dimensions see legend of suppl. Fig. S1a). Our calculation resembles the value of 169.1 mm^2^ reported by Roberts and Harrison^21^, which they calculated with different geometrical approximations for head and abdomen than in the present study.

### Simple mixed model

Using Eq. (7) to calculate the energy turnover of stationary foragers did not provide acceptable results (*h*_*b*_ = 12.14393 W m^−2^ °C^−1^, *r*_*l*_ = 0.68114, *r*_*g*_ = 0.30352, and *e*_*lev*_ = 1.82501). The SD of residuals (SD_res_) was only reduced to 15.3 mW (adjusted R^2^ = 0.24078, ANOVA), from a SD of 17.59 mW of the original data (Fig. 3a).

### Advanced mixed model

**Figure 4:**
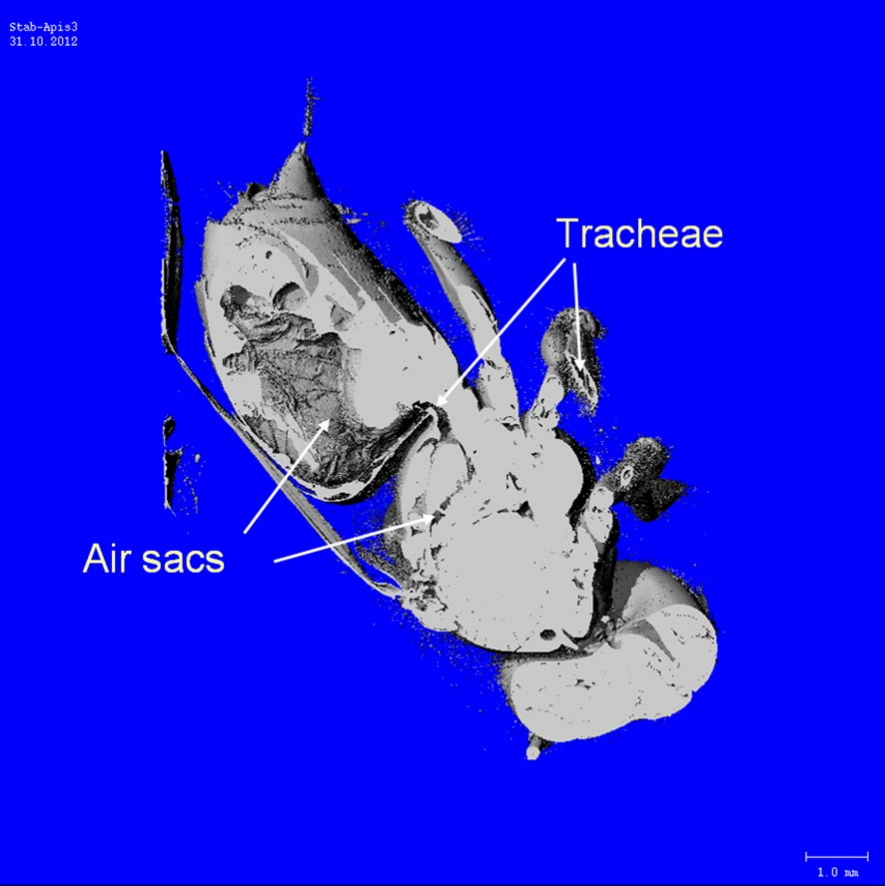
Micro computer tomogram (µCT) of a honeybee, showing abdominal and thoracic air sacs and tracheae of the tracheal system for respiratory heat exchange via internal convection and evaporation (microCT 40, SCANCO Medical, Bruettisellen, Switzerland). Please note that abdominal length is usually up to ~ 60% longer in living animals. See supplementary Video S1 for a slice-wise journey through the whole bee.

**Figure 5:**
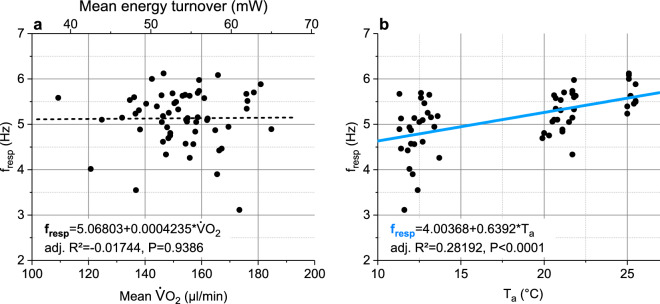
Dependence of stationary honeybee forager respiratory frequency (f_resp_) on (**a**) oxygen turnover (VO_2_), and (**b**) ambient air temperature (T_a_). Bees gathered 0.5 M or 1.5 M sucrose solution in unlimited flow. R^2^ = adjusted for degrees of freedom.

Calculating (or using) a fixed convection coefficient *h*_*b*_ during the fit procedure, despite the vast variation of environmental conditions (T_a_ = 14.8–37.5 °C, global radiation = 3.4–921 W m^−2^; means per stay at an artificial flower), was identified as a main factor hindering accurate prediction of metabolism from body temperature. *h*_*b*_ is made up of external (free or forced) and internal (respiratory) convection, the latter being of much greater importance in endothermic honeybee foragers than in ectothermic insects. However, during the experiments providing the dataset for model development^17,18^ we had not been able to measure the exact amount of external convection acting on the bees in the measurement chamber, and we do not know of a method to determine internal convective heat transport via tracheal respiration accurately in freely ranging bees. Calculation of a heat conductance estimate in shade (energy turnover/T_b_–T_a_, in mW °C^−1^) from the dataset revealed considerable changes not only with T_a_ but also with experimental conditions (e.g. unlimited or limited sucrose flow) (Fig. 3b; see also Ref.^17^). Foraging in the sun changed relations additionally (Fig. 3b). It has to be expected that internal convection via the honeybee tracheal system with its large air sacs (Fig. 4) makes up a considerable amount of convective heat loss because respiratory frequency had been found to amount to several Hz in foragers (Fig. 5; Ref.^7^) in comparison to only about 10–50 mHz in resting individuals^42^. Internal convection due to respiratory ventilation has to be expected to be quite small in the head but high in the thorax and the abdomen (Fig. 1b). Therefore, to improve accuracy, the convective heat exchange term (E_cv_ = *h*_*b*_ × A_b_(T_b_–T_a_)) had to be calculated independently for the three body parts, according to

12 $${\text{E}}_{cv} = h_{hd} \times {\text{A}}_{{{\text{hd}}}} \left( {{\text{T}}_{{{\text{hd}}}} - {\text{T}}_{{\text{a}}} } \right) \, + h_{th} \times {\text{A}}_{{{\text{th}}}} \left( {{\text{T}}_{{{\text{th}}}} - {\text{T}}_{{\text{a}}} } \right) + h_{ab} \times {\text{A}}_{{{\text{ab}}}} \left( {{\text{T}}_{{{\text{ab}}}} - {\text{T}}_{{\text{a}}} } \right).$$

**Figure 6:**
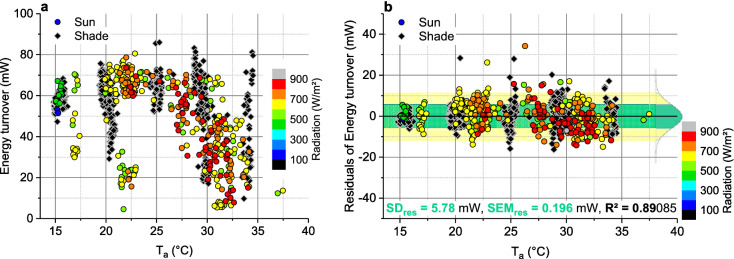
Energetics and model results of sucrose gathering honeybees foraging in shade (≤ 100 W m^−2^) or in sunshine (> 100 W m^−2^), in dependence on ambient air temperature (T_a_). (**a**) Energy turnover; original data of bees fed 1.5 M sucrose solution in unlimited flow (from^17^), and 0.5 M sucrose in unlimited flow or limited flow of 15 µl min^−1^ (from^18^); N = 872 visits to artificial flowers; SD = 17.59 mW. (**b**) Residuals of model calculation according to the use of Eq. (13) in Eq. (6), see Table 2a for model coefficients; green bar =  ± 1 SD_res_, light yellow bar =  ± 2 SD_res_, R^2^ = adjusted for degrees of freedom; residual standard error of mean (SEM_res_) = 0.196 mW.

Since most of the heat produced by honeybee foragers originates from the thorax (Fig. 1a,b), introduction of respiratory heat loss into the model might be improved by introducing *h*_*b*_ as a function of respiratory frequency (f_resp_), similar as was done by Henwood^34^ for changes of external convection due to the effect of wind. Our initial suggestion that f_resp_ might be a direct function of metabolism, because one would expect more heating activity to require a better oxygen supply of the thoracic flight muscles, was not supported by our measurements (Fig. 5a). There was no sign of a correlation between these variables but f_resp_ changed clearly with ambient air temperature (Fig. 5b)! From this finding that f_resp_ = *f*(T_a_) we concluded that *h*_*b*_ = *f*(T_a_). A possibility to introduce the dependence of convective heat loss on T_a_ into Eq. (12) is to introduce the convection coefficients of head, thorax and abdomen (*h*_*ca*_, *h*_*th*_, *h*_*ab*_) as a function of T_a_, making the convective heat exchange term take the form of

13 $${\text{E}}_{{{\text{cv}}}} = \left( {a_{hhd} + b_{hhd} \times {\text{T}}_{{\text{a}}} } \right) \times {\text{A}}_{{{\text{hd}}}} \left( {{\text{T}}_{{{\text{hd}}}} - {\text{T}}_{{\text{a}}} } \right) + \left( {a_{hth} + b_{hth} \times {\text{T}}_{{\text{a}}} } \right) \times {\text{A}}_{{{\text{th}}}} \left( {{\text{T}}_{{{\text{th}}}} - {\text{T}}_{{\text{a}}} } \right) + \left( {a_{hab} + b_{hab} \times {\text{T}}_{{\text{a}}} } \right) \times {\text{A}}_{{{\text{ab}}}} \left( {{\text{T}}_{{{\text{ab}}}} - {\text{T}}_{{\text{a}}} } \right),$$

the coefficients *a*_*hxx*_ and *b*_*hxx*_ being determined by numerical (iterative) calculation. Calculated coefficients can be found in Table 2a. This way, the accuracy of the model could be significantly improved, with SD_res_ = 5.78 mW (Fig. 6b) and the residual standard error of the mean (SEM_res_) being as small as 0.196 mW. This model variant explains ~ 89% of total variability (adjusted R^2^ = 0.89085; N = 872, df = 863; ANOVA).

**Table 2:** Regression coefficients for mixed, mechanistic and correlative, heat exchange models. (**a**) Model according to Eq. (13) in Eq. (6); ANOVA: F-value = 8638.83122, df = 863, *P* <<< 0.0001. (**b**) Simplified model according to Eq. (14) in Eq. (6); ANOVA: F-value = 9729.44757, df = 864, *P* <<< 0.0001. M = metabolic energy turnover, R_loss_ = radiative heat loss, R_gain_ = radiative heat gain, E_cv_ = convective heat exchange, E_ev_ = evaporative heat exchange (~ 0.004 W;^21,22^); A_hd_, A_th_, A_ab_ = surface area of head, thorax and abdomen, respectively; T_hd_, T_th_, T_ab_ = surface temperature of head, thorax and abdomen, respectively; T_a_ = ambient air temperature; SD_res_ = SD of residuals; adj. R^2^ = adjusted for df. *P* >|*t*| provides a measure of the statistic relevance of a coefficient.

M = *r*_*l*_ × R_loss_ − *r*_*g*_ × R_gain_ + E_cv_ + *e*_*lev*_ × E_ev_ [W]; see Eq. (6)							
a) E_cv_ = (*a*_*hhd*_ + *b*_*hhd*_ × T_a_) × A_hd_(T_hd_–T_a_) + (*a*_*hth*_ + *b*_*hth*_ × T_a_) × A_th_(T_th_–T_a_) + (*a*_*hab*_ + *b*_*hab*_ × T_a_) × A_ab_(T_ab_–T_a_); see Eq. (13)				b) E_cv_ = (*a*_*hhd*_ + *b*_*hhd*_ × T_a_) × A_hd_(T_hd_–T_a_) + (*a*_*hth*_ + *b*_*hth*_ × T_a_) × A_th_(T_th_–T_a_) + *h*_*ab*_ × A_ab_(T_ab_–T_a_); see Eq. (14)			
SD_res_ = 0.0578 W; adj. R^2^ = 0.89085; N = 872				SD_res_ = 0.0578 W; adj. R^2^ = 0.89097; N = 872			
Coefficient	Value	*t* value	*P* >|*t*|	Coefficient	Value	*t* value	*P* >|*t*|
*r*_*l*_	0.33306	1.81631	0.06967	*r*_*l*_	0.33816	1.86167	0.06299
*r*_*g*_	0.06799	5.28299	1.61E-07	*r*_*g*_	0.06758	5.31464	1.36E-07
*e*_*lev*_	− 8.61143	− 2.4425	0.01479	*e*_*lev*_	− 7.80027	− 10.66662	0
*a*_*hhd*_	51.73447	1.94636	0.05194	*a*_*hhd*_	55.80168	3.0797	0.00214
*b*_*hhd*_	− 7.62225	− 6.79265	2.05E-11	*b*_*hhd*_	− 8.71008	− 2.49426	0.01281
*a*_*hth*_	26.88355	3.15739	0.00165	*a*_*hth*_	26.57184	3.17143	0.00157
*b*_*hth*_	5.26661	15.43998	0	*b*_*hth*_	5.28199	15.86683	0
*a*_*hab*_	− 21.14844	− 2.80945	0.00507	*h*_*ab*_	− 22.66916	− 11.56947	0
*b*_*hab*_	− 0.06052	− 0.20924	0.83431	–	–	–	–

However, a subsequent analysis about the effect of individual predictors on the dependent variable (evaluating their relative “importance”), via the estimated standard errors of the regression coefficients and the associated t-test probabilities^43,44^, revealed the coefficient *b*_*hab*_ to contribute quite insignificantly to the regression (*P* >|t|= 0.8341; Table 2; compare^41^). This may be due to the fact that *h*_*ab*_ calculated according to *h*_*ab*_ = (*a*_*hab*_ + *b*_*hab*_ × T_a_) from Eq. (13) changed only marginally with T_a_ (see suppl. Fig. S4). Simplifying Eq. (13) to

14 $${\text{E}}_{{{\text{cv}}}} = \, \left( {a_{hhd} + b_{hhd} \times {\text{T}}_{{\text{a}}} } \right) \times {\text{A}}_{{{\text{hd}}}} \left( {{\text{T}}_{{{\text{hd}}}} - {\text{T}}_{{\text{a}}} } \right) + \left( {a_{hth} + b_{hth} \times {\text{T}}_{{\text{a}}} } \right) \times {\text{A}}_{{{\text{th}}}} \left( {{\text{T}}_{{{\text{th}}}} - {\text{T}}_{{\text{a}}} } \right) + h_{ab} \times {\text{A}}_{{{\text{ab}}}} \left( {{\text{T}}_{{{\text{ab}}}} - {\text{T}}_{{\text{a}}} } \right),$$

led to a quite similar prediction accuracy (SD_res_ = 5.78 mW; adj. R^2^ = 0.89097). For coefficients see Table 2b. All attempts to eliminate further coefficients or predictor variables from the model led to a decreased accuracy.

### Advanced three-compartment model

Convection, however, does not only occur between the bee body and the adjacent air. Forced convection by haemolymph circulation between body parts also transfers considerable amounts of heat, not only from the thorax (the main heat source) to head and abdomen but also back from the cooler body parts to the thorax (Fig. 1b). A “three-compartment model” approach^45^, replacing the temperature excess of the body parts head and abdomen to ambient air temperature (T_hd_–T_a_ and T_ab_–T_a_) by the difference between the thorax and these body parts (T_th_–T_hd_ and T_th_–T_ab_), respectively, with

15 $${\text{E}}_{{{\text{cv}}}} = \left( {a_{hthhd} + b_{hthhd} \times {\text{T}}_{{\text{a}}} } \right) \times \left( {{\text{T}}_{{{\text{th}}}} - {\text{T}}_{{{\text{hd}}}} } \right) + \left( {a_{hth} + b_{hth} \times {\text{T}}_{{\text{a}}} } \right) \times {\text{A}}_{{{\text{th}}}} \left( {{\text{T}}_{{{\text{th}}}} - {\text{T}}_{{\text{a}}} } \right) + \left( {a_{hthab} + b_{hthab} \times {\text{T}}_{{\text{a}}} } \right) \times \left( {{\text{T}}_{{{\text{th}}}} - {\text{T}}_{{{\text{ab}}}} } \right)$$

led to the same accuracy, with SD_res_ = 5.78 mW and adj. R^2^ = 0.89085. For coefficients see Table 3a.

**Table 3:** Regression coefficients for mixed three compartment heat exchange models (comp. Stavenga et al.^45^). (**a**) Model according to Eq. (15) in Eq. (6); ANOVA: F-value = 8638.83122, df = 863, *P* <<< 0.0001. (**b**) Simplified model according to Eq. (16) in Eq. (6); ANOVA: F-value = 9729.44757, df = 864, *P* <<< 0.0001. M = metabolic energy turnover, R_loss_ = radiative heat loss, R_gain_ = radiative heat gain, E_cv_ = convective heat exchange, E_ev_ = evaporative heat exchange (~ 0.004 W;^21,22^); A_hd_, A_th_, A_ab_ = surface area of head, thorax and abdomen, respectively; T_hd_, T_th_, T_ab_ = surface temperature of head, thorax and abdomen, respectively; T_a_ = ambient air temperature; SD_res_ = SD of residuals; adj. R^2^ = adjusted for df. *P* >|*t*| provides a measure of the statistic relevance of a coefficient.

M = *r*_*l*_ × R_loss_ − *r*_*g*_ × R_gain_ + E_cv_ + *e*_*lev*_ × E_ev_ [W]; see Eq. (6)							
a) E_cv_ = (*a*_*hthhd*_ + *b*_*hthhd*_ × T_a_) × (T_th_–T_hd_) + (*a*_*hth*_ + *b*_*hth*_ × T_a_) × A_th_(T_th_–T_a_) + (*a*_*hthab*_ + *b*_*hthab*_ × T_a_) × (T_th_–T_ab_); see Eq. (15)				b) E_cv_ = (*a*_*hthhd*_ + *b*_*hthhd*_ × T_a_) × (T_th_–T_hd_) + (*a*_*hth*_ + *b*_*hth*_ × T_a_) × A_th_(T_th_–T_a_) + *h*_*thab*_ × (T_th_–T_ab_); see Eq. (16)			
SD_res_ = 0.0578 W; adj. R^2^ = 0.89085; N = 872				SD_res_ = 0.0578 W; adj. R^2^ = 0.89097; N = 872			
Coefficient	Value	*t* value	*P* >|*t*|	Coefficient	Value	*t* value	*P* >|*t*|
*r*_*l*_	0.33306	1.81631	0.06967	*r*_*l*_	0.33816	1.86167	0.06299
*r*_*g*_	0.06799	5.28299	1.61E-07	*r*_*g*_	0.06758	5.31464	1.36E-07
*e*_*lev*_	− 8.61143	− 2.4425	0.01479	*e*_*lev*_	− 8.71008	− 2.49426	0.01281
*a*_*hthhd*_	− 0.00155	− 1.94636	0.05194	*a*_*hthhd*_	− 0.00167	− 3.0797	0.00214
*b*_*hthhd*_	2.29E-04	6.79265	2.05E-11	*b*_*hthhd*_	2.34E-04	10.66662	0
*a*_*hth*_	21.34836	3.72077	2.11E-04	*a*_*hth*_	20.85664	3.98683	7.26E-05
*b*_*hth*_	0.37104	1.53302	0.12564	*b*_*hth*_	0.38344	1.63488	0.10244
*a*_*hthab*_	0.00182	2.80945	0.00507	*h*_*thab*_	0.00195	11.56947	0
*b*_*hthab*_	5.20E-06	0.20924	0.83431	–	–	–	–

Again, simplifying Eq. (15) to

16 $${\text{E}}_{{{\text{cv}}}} = \, \left( {a_{hthhd} + b_{hthhd} \times {\text{T}}_{{\text{a}}} } \right) \times \left( {{\text{T}}_{{{\text{th}}}} - {\text{T}}_{{{\text{hd}}}} } \right) + \left( {a_{hth} + b_{hth} \times {\text{T}}_{{\text{a}}} } \right) \times {\text{A}}_{{{\text{th}}}} \left( {{\text{T}}_{{{\text{th}}}} - {\text{T}}_{{\text{a}}} } \right) + h_{thab} \times \left( {{\text{T}}_{{{\text{th}}}} - {\text{T}}_{{{\text{ab}}}} } \right)$$

resulted in a similar accuracy, with SD_res_ = 5.78 mW and adj. R^2^ = 0.89097. For coefficients see Table 3b.

## Discussion

In ecology, animal energetic modelling may be mechanistic, building solely on physical and physiological input, or correlative, using known relationships without detailed knowledge of all underlying mechanisms and the exact magnitude of factors^32^. The mechanistic approach requires more investment of time and resources to determine the underlying causal processes in detail^28,32^. The alternative correlative approach usually saves resources and time and often may provide faster and more accurate results^32^. Mechanistic models are thought to be better transferable to different environmental conditions than correlative ones. The accuracy of such models, however, depends strongly on the exact knowledge of heat exchange parameters, which may change with foraging condition in honeybees (Fig. 3b; compare^17,18,22^). We therefore take a stand for mixed models, based on sufficient experimental datasets and proper consideration of physics and physiology but correlative weighting of parameters where necessary.

Heat exchange, for example, does not only occur between the insect body and the environment but also between body parts by two mechanisms: forced convection via haemolymph circulation and respiration (Fig. 1). Abdominal volume, and thus surface for radiative and convective heat exchange, usually changes during foraging because a bee can load nectar of nearly the own weight during a foraging trip^46^, and body postures relative to the sun may change (compare suppl. Fig. S3). Physiological needs and effects of environmental variation on body function have to be considered and verified by experiments ^8^, because physiological regulatory mechanisms are complex and hardly predictable from physical assumptions. The interrelation between physical mechanisms and physiological regulation is often not clearly accessible. The surprising finding that respiratory frequency (f_resp_), as a correlative of (internal) respiratory convection, correlated with ambient air temperature (T_a_) but not with oxygen consumption (Fig. 5) underpins the need of an experimental foundation for theoretical considerations. We suggest this change with T_a_ to originate from the change of the function of the abdominal muscles for tracheal ventilation with T_a_ (compare^47,48^), because abdominal temperature follows T_a_ more closely than the temperature of other body parts (e.g.^7,18^).

A main factor influencing heat loss of endothermic animals is the difference of body temperature to ambience (T_b_–T_a_; e.g.^24,26,27,34,45,49^). In honeybees heating their thorax up in flight preparation, a rather straight correlation between oxygen turnover and T_thorax_–T_a_ has been reported^50^. In thermoregulating bees in thermal steady state, however, this relationship is not a simple one (compare Fig. 6a). The relation changes strongly with ambient temperature, radiation and foraging condition, and shows a huge variability (Fig. 3a, suppl. Fig. S2; compare^17,18^). Figure 3b shows that in stationary endothermic honeybees the simple calculation of a heat conductance estimate (mW °C^−1^) delivers considerably differing results for bees drinking sucrose solution in unlimited and limited flow. Foraging in sunshine provides even more variation because foragers can use solar heat gain to save energy or to speed up foraging^17,18^. Therefore, since honeybees *must not* be treated as simple physical objects but have many possibilities of physiological and behavioural reaction to environmental variation (Fig. 1;^6,8,9,17,18,21,22^), the accuracy of purely mechanistic models of heat exchange will remain limited. The solution are mixed models as presented here, integrating correlative and mechanistic approaches^32^. Our model variants use physical input of relevant heat exchange parameters but a correlative weighting of this input via the calculated coefficients. The correlative (iterative) determination of multiple coefficients, however, implies that they are not determined completely independent from each other when the underlying algorithm is searching for the optimal fit (Tables 2, 3)^44^. Therefore, always the full coefficient set is necessary for best predictions. Since honeybees own a variety of physiological and behavioural mechanisms to regulate heat flow inside the body and heat exchange with the environment (Fig. 1b), heat exchange coefficients calculated correlatively may differ from mere static physical calculations, and even may take ‘unexpected’ values. The negative values calculated for head (*h*_*hd*_) and abdomen (*h*_*ab*_) as drawn in supplementary Fig. S4 (calculated according to Table 2a) may be interpreted in a way that these body parts on average receive more heat than they emit to the environment, from the thorax via haemolymph circulation and tracheal ventilation, and from the sun (compare Fig. 1). A different coefficient set will have to be determined for bees in free flight, because the relation between external convection by headwind and wing movement, and internal convection by respiration and possibly blood flow, will change.

We tried to build our model variants as detailed as necessary for high prediction accuracy but keep them as simple as possible to achieve a high practical usability. The great advantage of our mixed approach is its high and proven accuracy, which is quite similar throughout most of the natural variation of the environmental factors ambient air temperature and global radiation (Fig. 6b). The model variants allow estimation of energetic costs from measurements of body (surface) temperature of bees foraging for example on water sources and honeydew droplets, and on many types of flowers where bees remain stationary for some time. This kind of flowers includes composite plants like dandelion (*Taraxacum* sp.), sunflower (*Helianthus* sp.), thistle (*Cirsium* sp.), and some stonecrops (*Sedum* sp.), etc. For bees foraging in shade with more frequent and longer flights between flowers, for example on plants like apricot (*Prunus* sp.) or raspberry (*Rubus* sp.), the laboratory measurements of bees hovering or flying in a measurement chamber^21,22^ may provide a preliminary approximation of energy turnover. However, to provide best results, these measurements have to be repeated under outdoor conditions (see^22^) which include measurement of ambient air temperature and global (solar) radiation in addition to respiration and body temperature. Tracking of honeybee flights in flower patches (e.g.^51–55^) will help to better quantize the relative amounts of stationary thermoregulation and flight. For longer lasting free flights out to a flower patch and back, outdoor measurements (e.g.^22^) will allow a preliminary estimation of energy costs in shade if flight time is known. Again, for best results simultaneous measurement of respiration and body temperature in combination with relevant environmental parameters is necessary to deliver best results for energetic modelling of honeybee foraging.

## Conclusion

With a mixed model approach of honeybee heat exchange, integrating the benefits of mechanistic and correlative models, we were able to predict the energy turnover of stationary honeybee foragers from measurements of body (surface) temperature and basic environmental parameters. The model provides high and proven accuracy throughout the main ranges of environmental variation bees usually experience during foraging, of ambient air temperature ~ 14–38 °C, and of global radiation ~ 3–1000 W m^−2^.

## Supplementary Information

Supplementary Information 1.

Supplementary Video 1.

## Data Availability

The datasets used and/or analysed during the current study are available from the corresponding author on reasonable request.
