# Supplementary material for: A mixed model of heat exchange in stationary honeybee foragers
Source: Sci Rep. 2023 Mar 21;13:4655. doi: 10.1038/s41598-023-31320-5 (PMC10030634; doi:10.1038/s41598-023-31320-5)
Supplement: Supplementary file 1 — Supplementary Information 1. [file 41598_2023_31320_MOESM1_ESM.pdf]

Supplementary Information:

A mixed model of heat exchange in stationary honeybee foragers

Anton Stabentheiner[a1] & Helmut Kovac[a1]

[a]Institute of Biology, University of Graz, Universitätsplatz 2, 8010 Graz, Austria.

[1]To whom correspondence may be addressed. E-mail: anton.stabentheiner@uni-graz.at or helmut.kovac@uni-graz.at.

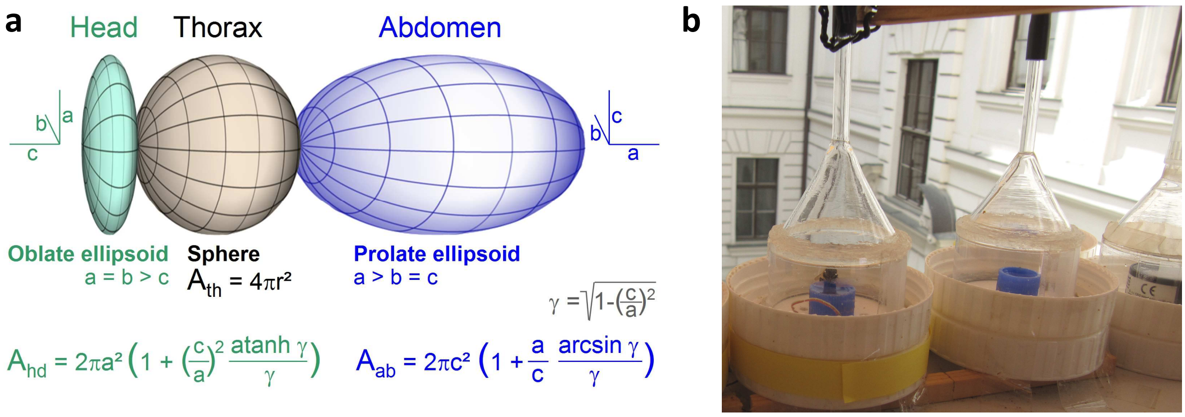

Figure S1. (**a**) Body surface modelling in foraging honeybees. Body dimensions for body surface calculation: head (A_{hd}), a = b = 0.85 mm, c = 1.9 mm; thorax (A_{th}), r = 1.95 mm; abdomen (A_{ab}), a = 4 mm, b = c = 2 mm.
Ellipsoids and sphere adapted from: https://en.wikipedia.org/wiki/File:ProlateSpheroid.png. (**b**) Measurement chamber (left) and reference chamber (right) for simultaneous measurement of O_2 consumption and respiratory frequency (f_{resp}).

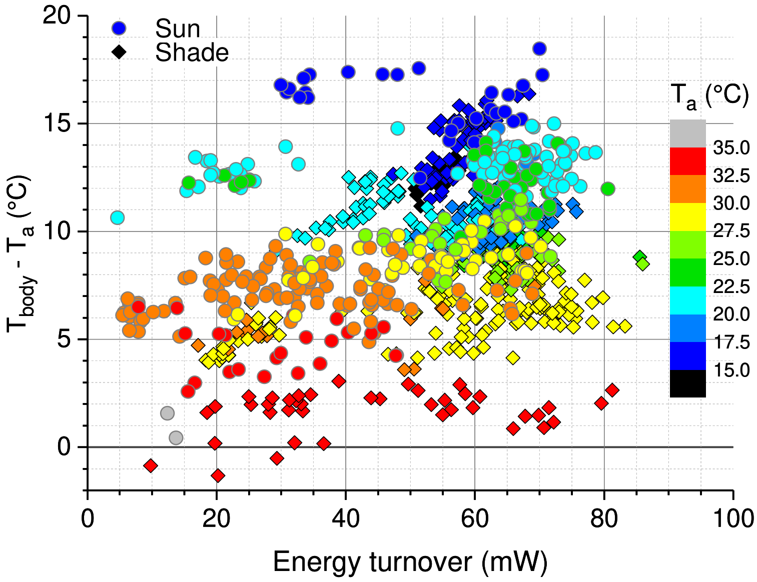

Figure S2. Body surface temperature excess T_{body}-T_a (elevation of mean of head (T_{hd}), thorax (T_{th}) and abdomen (T_{ab}) above ambient air temperature T_a) in dependence on energy turnover of sucrose gathering honeybees. Shade ≤ 100 W m^{-2}, sunshine > 100 W m^{-2}. Bees fed 1.5 M sucrose in unlimited flow (from Stabentheiner and Kovac 2014) [17], and 0.5 M sucrose solution in unlimited flow or limited flow of 15 µl min^{-1} (from Stabentheiner and Kovac 2016) [18].

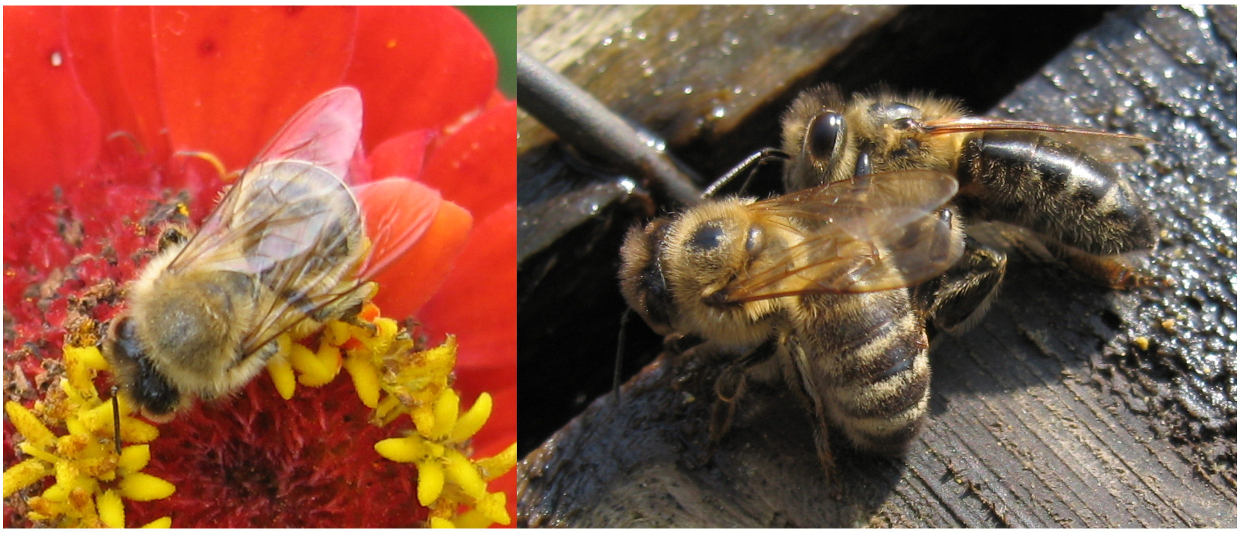

Figure S3. Honeybees foraging on a flower or a water source. Note possible differences in thoracic hair cover (especially on thorax and head), of body postures due to bending of the abdomen, and changing absorbing body surface area because of differing positions relative to the sun. The hairs not only reduce convective heat loss (see Heinrich 1993) [10] but are probably also important in reducing absorption of solar heat, and they influence radiative and convective heat exchange. Moreover, this hairy 'pile' may change considerably with age, abrasion occurring with time especially in foragers. In addition, absorption and reflection by wings, and variation of visual absorptivity across the body and between individuals influence radiative heat exchange.

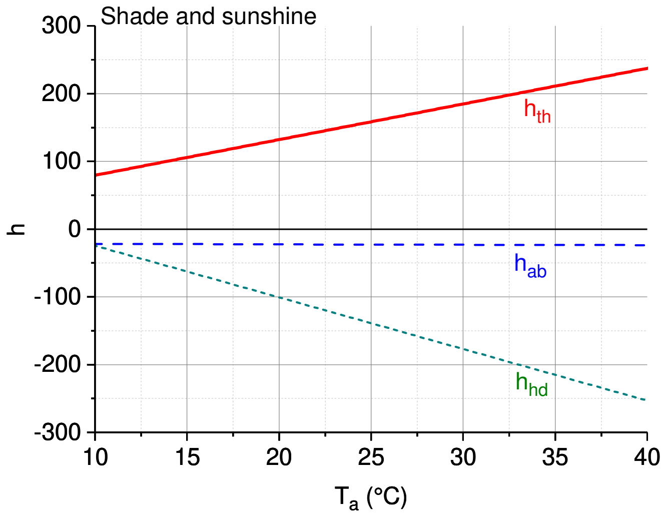

Figure S4. Calculated change of heat exchange coefficients h [W m^{-2} °C^{-1}] with T_a for head (h_{hd}), thorax (h_{th}) and abdomen (h_{ab}), of the model variant in Table 2a, according to equation (13) in equation (6).

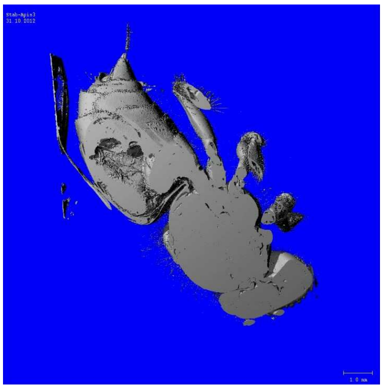

Video S1. Micro computer tomogram (µCT) journey through a honeybee, showing abdominal and thoracic air sacs, and the tracheal system: Video_Stab_Apis3_S.mov (duration: 1'20''). See also Fig. 4.
